# Estimation of Trunk Muscle Forces Using a Bio-Inspired Control Strategy Implemented in a Neuro-Osteo-Ligamentous Finite Element Model of the Lumbar Spine

**DOI:** 10.3389/fbioe.2020.00949

**Published:** 2020-08-11

**Authors:** Alireza Sharifzadeh-Kermani, Navid Arjmand, Gholamreza Vossoughi, Aboulfazl Shirazi-Adl, Avinash G. Patwardhan, Mohamad Parnianpour, Kinda Khalaf

**Affiliations:** ^1^Department of Mechanical Engineering, Sharif University of Technology, Tehran, Iran; ^2^Division of Applied Mechanics, Department of Mechanical Engineering, Polytechnique Montréal, Montreal, QC, Canada; ^3^Musculoskeletal Biomechanics Laboratory, Edward Hines, Jr. VA Hospital, Hines, IL, United States; ^4^Department of Biomedical Engineering, Khalifa University of Science and Technology, Abu Dhabi, United Arab Emirates

**Keywords:** spine, model, controller, muscle force, follower load, stability

## Abstract

Low back pain (LBP), the leading cause of disability worldwide, remains one of the most common and challenging problems in occupational musculoskeletal disorders. The effective assessment of LBP injury risk, and the design of appropriate treatment modalities and rehabilitation protocols, require accurate estimation of the mechanical spinal loads during different activities. This study aimed to: (1) develop a novel 2D beam-column finite element control-based model of the lumbar spine and compare its predictions for muscle forces and spinal loads to those resulting from a geometrically matched equilibrium-based model; (2) test, using the foregoing control-based finite element model, the validity of the follower load (FL) concept suggested in the geometrically matched model; and (3) investigate the effect of change in the magnitude of the external load on trunk muscle activation patterns. A simple 2D continuous beam-column model of the human lumbar spine, incorporating five pairs of Hill’s muscle models, was developed in the frontal plane. Bio-inspired fuzzy neuro-controllers were used to maintain a laterally bent posture under five different external loading conditions. Muscle forces were assigned based on minimizing the kinematic error between target and actual postures, while imposing a penalty on muscular activation levels. As compared to the geometrically matched model, our control-based model predicted similar patterns for muscle forces, but at considerably lower values. Moreover, irrespective of the external loading conditions, a near (<3°) optimal FL on the spine was generated by the control-based predicted muscle forces. The variation of the muscle forces with the magnitude of the external load within the simulated range at the L1 level was found linear. This work presents a novel methodology, based on a bio-inspired control strategy, that can be used to estimate trunk muscle forces for various clinical and occupational applications toward shedding light on the ever-elusive LBP etiology.

## Introduction

Low back pain (LBP) as the leading cause for work loss and years lived with disability emerges also as the most common and costliest problem in occupational musculoskeletal disorders (Clark and Horton, 2018; Hartvigsen et al., 2018). In the United States alone, the annual cost of LBP was estimated at ∼$200 billion in 2006 (Katz, 2006). This asserts the important role of biomechanical investigations to mitigate and manage the associated risk of injury through quantitative assessment of the mechanical loads on the spine during various daily and occupational activities. In the absence of adequate non-invasive *in vivo* measurement techniques, a number of musculoskeletal spine models, with different degrees of complexities, have been developed to estimate the internal loads in the active-passive structures of the trunk (Dreischarf et al., 2016; Ghezelbash et al., 2020). Due to the large number of trunk muscles spanning the intervertebral joints, the available equations are insufficient to solve this mechanically indeterminate system toward a unique solution, i.e., joint kinetics redundancy. The kinematic redundancies in the multi-joint spinal column, while providing flexibility in performing a specific task, add further complexity to the motor control strategies (Parnianpour, 2013). They can be viewed as the abundance to manage the conflicting objectives due to alterations in the environmental conditions and/or changes in task demand priorities (Latash et al., 2010).

Two distinct approaches are generally used to resolve the redundancies in such musculoskeletal models: inverse (e.g., equilibrium- and equilibrium-stability-based) and forward (e.g., control-based) dynamic. Equilibrium-based models leverage the available kinematics and governing equilibrium equations at various levels/joints/directions, and employ an optimization algorithm [often combined with limited recording of surface muscle electromyography (EMG)], to compute muscle forces and internal loads (Cholewicki and McGill, 1996; Parnianpour et al., 1997; Sparto and Parnianpour, 1998; Gagnon et al., 2011; Mohammadi et al., 2015; Dreischarf et al., 2016). In these models, the system, maintains equilibrium (static and/or dynamic) with no attention to crucial stability requirements. Imposing stability, in addition to the equilibrium, has led to the development of multi-criteria equilibrium-stability-based models, in which the kinetics redundancy can once again be resolved either by using an optimization/control theory-based algorithm (Hemami and Katbab, 1982; Granata and Orishimo, 2001; Zeinali-Davarani et al., 2008; Vakilzadeh et al., 2011; Hajihosseinali et al., 2014), or an EMG-driven algorithm (Samadi and Arjmand, 2018). The stability criterion in these models is typically investigated through the positive definiteness of the Hessian matrix of the system’s potential energy (Crisco and Panjabi, 1991; Cholewicki and McGill, 1996; Shirazi-Adl et al., 2005), or equivalently by the eigenvalues of the dynamic system (Hemami and Katbab, 1982; Bazrgari et al., 2008, 2009; Zeinali-Davarani et al., 2008; Shahvarpour et al., 2015). In general, considering stability requirements when calculating trunk muscle forces yields stronger correlation between predicted muscle activation and experimentally measured EMG data (Granata and Orishimo, 2001; Hajihosseinali et al., 2014; Samadi and Arjmand, 2018).

Unlike the inverse dynamics approaches, forward control-based dynamic models assign forces to muscles, either individually or synergistically grouped, in alignment with the central nervous system’s (CNS) neural control strategies applied in trunk movements. The controller used in these models commonly adjusts muscle forces in search of target postural trajectories, while maintaining dynamic equilibrium and stability requirements (Dariush et al., 1998). Predictions of control-based models have been successfully validated against EMG data (Sedighi et al., 2011; Nasseroleslami et al., 2014). Due to the challenging geometrical complexity and intricate multi-joint structures in the human trunk, previous control-based models have mainly simplified the upper trunk as an inverted pendulum with a single ball-and-socket (spherical) joint fixed at its base [i.e., the lumbosacral (L5/S1) junction]. This approach neglects relative deformations at the upper levels, translational degrees of freedom (DoFs), and changes in the centers of rotation (CoRs) under varying motions/loading conditions (Nasseroleslami et al., 2014). Recent investigations have demonstrated the variable effects of both the joint positioning (Ghezelbash et al., 2018) and joint translational DoFs (Cashaback et al., 2013; Ghezelbash et al., 2015) on the kinematics, as well as, muscle forces and spinal loads. While a control-based model of the whole body is used to provide more geometrical details, it is based on multi-body simulations of the spine thus neglecting the intervertebral joint complexities (Rupp et al., 2015). Up to date, however, only one control-based FE model of the entire body included translational DoFs (with movement restricted to the sagittal plane), while using a simplistic proportional-integral-derivative (PID) controller, to determine muscle activations (Östh, 2010; Andersson, 2013). To provide more geometrical details, deformable elements, based on fitting a curve on the forces and moments previously obtained by a finite element model of the intervertebral disc, were added to the multi-body model of the lumbar spine; again neglecting the intervertebral joint complexities (Karajan et al., 2013, 2014; Rupp et al., 2015). Moreover, a previous study included active muscle models in a reduced musculoskeletal finite element model of the lumbar spine to explore possible functional relationships between muscle function and intervertebral disc condition (Toumanidou and Noailly, 2015).

The objectives of the present study are as follows:

(1)To develop a novel 2D beam-column control-based model of the lumbar spine and compare its muscle force predictions with an existing geometrically matched equilibrium-based model (Patwardhan et al., 2001). The model incorporate (1) the DoFs at all levels of the spine [via implementing the controller in a finite element model of plant (passive spine)] thus also approximating changes in the joint CoRs, (2) force-length and force-velocity relationships in muscles using a Hill-based muscle model (Zajac, 1989), and (3) a bio-inspired control strategy to estimate muscle forces using fuzzy neuro-controllers with an emotional learning algorithm that adequately mimics the adaptive mechanism of the CNS. The controller minimizes kinematic deviations between actual and target postures, while calculating muscle activations by penalizing the controller unit for muscle activation level (Nasseroleslami et al., 2014).

(2)To investigate, using the foregoing control-based finite element model, the follower load (FL) concept as suggested in the geometrically matched equilibrium-based model (Patwardhan et al., 2001). In that model, the muscle forces were estimated based on the premise that the resultant compressive load on the spine behaves as a follower load (FL) (i.e., a load that follows the curvature of the lumbar spine, at all lumbar levels and postures), thus providing inherent spinal stability, as observed in *in vitro* studies. This strategy implicitly leverages the stability requirement by minimizing horizontal translations/rotations along the spine. We hypothesize that our control strategy (selected to mimic the role of the CNS in resolving the kinetic redundancy) automatically leads to trunk muscle forces consistent with a FL on the spine, thereby maximizing the mechanical stability of the spine. This suggests that the controller used in our model would learn to activate muscles in a manner that not only minimizes the kinematic deviations, but also the destabilizing shear forces and moments.

(3)To investigate the effect of external load magnitude on the trunk muscle activation patterns. It is hypothesized that the predicted pattern of muscle activation is scaled with the external load magnitude, thus providing evidence for a synergistic activation.

## Materials and Methods

### Geometry and Musculature of the Lumbar Spine Model

**FIGURE 1:**
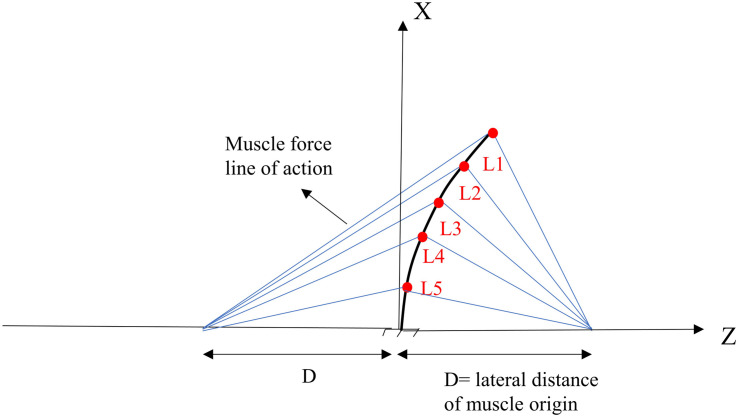
Geometry and musculature of the lumbar spine model in a laterally flexed posture in the frontal plane (Patwardhan et al., 2001).

For the sake of comparison and hypothesis testing, the geometry of our deformable beam-column model of the lumbar spine and musculature were selected to be identical to those introduced in a previous work (Patwardhan et al., 2001). A simple 2D model of the lumbar spine, as a continuous elastic beam-column in the frontal plane, was constructed in LS-DYNA^®^ (Livermore Software Technology Corporation, Livermore, CA, United States) (Figure 1). Five distinct pairs of muscles were attached to a fixed base (representing the pelvis/sacrum) of the deformable beam at various L1–L5 lumbar levels. The simulation at the steady-state condition was quasi-static; upper body masses and inertias were hence neglected. The gravitational effect of masses was, however, accounted for by either a concentrated force at the L1 or distributed forces at various nodes (Table 1). The exact beam geometry, flexural rigidity (EI = 1.9 Nm^2^), and coordinates of upper/lower muscle insertions were all adopted from earlier work (Table 2; Patwardhan et al., 2001). The cross sectional area of the column was assumed constant at 1225 mm^2^. The model was fixed at the sacrum (lower node) and restricted elsewhere to solely move in the frontal plane. The lumbar spine model consisted of five Hughes-Liu beam elements. The Hughes-Liu beam is a degenerated 8-node solid element (linear displacement and rotation field) with high computational efficiency and robustness (Hallquist, 2006). Sensitivity of the model predictions to the number of beam elements in the model (i.e., mesh refinement) was verified.

**TABLE 1:** Simulation cases.

**Loading case**	***P*_*1*_ (N)**	***P*_*2*_ (N)**	***P*_*3*_ (N)**	***P*_*4*_ (N)**	***P*_*5*_ (N)**	**D (mm)**
1	635	0	0	0	0	50
2	350	50	50	50	50	50
3	110	110	110	110	110	50
4	110	110	110	110	110	25
5	110	110	110	110	110	75

***P*_*i*_ represents the gravitational force of the upper trunk acting on the lumbar vertebra *L*_*i*_. Gravitational loads increase from zero to *P*_*i*_ during 0.2 s. D represent the lateral distance of muscle origins.**

**TABLE 2:** Nodal coordinates of the deformed lumbar spine model (see Figure 1).

	**L1**	**L2**	**L3**	**L4**	**L5**
X (mm)	190	150	105	80	38
Z (mm)	10.0	6.2	3.0	1.7	0.4

### Hill’s Muscle Model

A Hill muscle model (Zajac, 1989) is used as follows:

(1) $$F=f_{\max}\left(\alpha .f_{l}\left(l\right).f_{v}\left(\dot{l}\right)+f_{p}\left(l\right)\right)$$

(2) $$f_{l}\,\left(l\right)=5.1-29\,\left(\frac{l}{l_{0}}\right)+56\,{\left(\frac{l}{l_{0}}\right)}^{2}-41\,{\left(\frac{l}{l_{0}}\right)}^{3}+10\,{\left(\frac{l}{l_{0}}\right)}^{4}$$

(3) $$f_{v}\,\left(\dot{l}\right)=\frac{0.1433}{0.1074+\exp\left(-1.409\,\sinh\left(3.2\,\frac{\bar{l}}{{\bar{l}}_{\max}}+1.6\right)\right)}$$

(4) $$f_{p}\,\left(l\right)=\exp\left(-10.671+7.675\,\frac{l}{l_{0}}\right)$$

In the above equations, *F*, *f _l_* (*l*) (Nussbaum and Chaffin, 1998), f_v_⁢(l) (Hatze, 1977), and *f*_*p*_(*l*) (McGill and Norman, 1986) are muscle force, as well as force-length, force-velocity and passive force-length relationships, respectively. Moreover, *f_max_*, α, *l*, $\dot{l}$, *l*_0_, ${\dot{l}}_{\max}$ represent muscle maximum force, muscle activation level, muscle length, muscle velocity, muscle resting length and maximum muscle velocity, respectively. The value of ${\dot{l}}_{\max}=\frac{l_{0}}{0.1\,s}$ is assumed in this study (Zajac, 1989). *f_max_* is assumed to be 800 N in all muscles. In muscles, the damping is represented intrinsically by the force-velocity relationship (Eq. 3), while the stiffness alters with the current length according to the force-length relationships (Eqs. 2 and 4). Inspection of the Hill type muscle response used (Eq. 1) reveals that the muscle activation affects the system response by modulating the muscle force and stiffness (Hogan, 1990). The spine structural stiffness matrix consists of contributions from both active and passive systems.

### Controller

**FIGURE 2:**
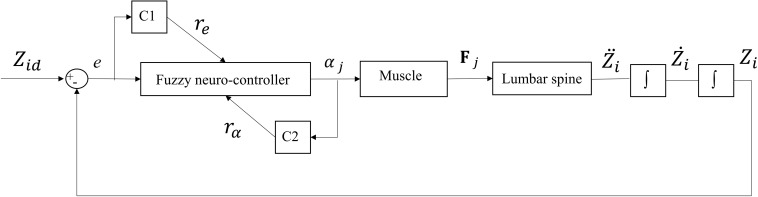
Feedback control loop with SISO fuzzy neuro-controller unit. C1 and C2 are the critics of the system that generate $r_{e}=h_{1}\,e+h_{2}\,\dot{e}+h_{3}\,\int e$ and *r*_α_ = abs(α), respectively. α_*j*_ is the level of activation of muscle (j) attached to *L*_*i*_ and *e* is the difference between desired (*Z_id_*) and actual (*Z*_*i*_) Z-coordinate of the node *L*_*i*_ (Figure 1).

One single-input and single-output (SISO) controller for each muscle was used to control the continuous beam model. The main idea behind the control structure assumes that each pair of bilateral muscles attached to a particular point increases the active stiffness at that point. The SISO controller used in this study is a fuzzy neuro-controller whose weights are tuned according to two critic signals (Figure 2; Lucas et al., 2004). The purpose of the controller is to minimize the general error function displayed below (Eq. 5) with the steepest descent algorithm:

(5) $$E=E_{e}+E_{\alpha }=\frac{1}{2}\,k_{e}\,{\left(h_{1}\,e+h_{2}\,\dot{e}+h_{3}\,\int e\right)}^{2}+\frac{1}{2}\,k_{\alpha }\,{\left(\mathrm{abs}\,\left(\alpha \right)\right)}^{2}$$

In the above equation, *e*, $\dot{e}$, and ∫*e*represent the error (difference between the actual and target kinematics), error rate, and the integral of error, where *h*_1_, *h*_2_, and *h*_3_ represent error, error rate and error integral coefficients. Moreover, *k*_*e*_ and *k*_α_ represent the weighting functions for the priority of the error signal components. α is the level of muscle activation (between 0 and 1). As can be seen in Eq. 5, the error function consists of two parts *E_e_*and *E*_α_,where*E_e_*represents the kinematics error, while *E*_α_penalizes the controller for the control activation signal and plays an essential role in resolving the system redundancy in terms of muscle forces (Nasseroleslami et al., 2014). The above cost function is defined for each muscle, where the error terms are based on the Z-coordinates of the nodes to which muscles are attached. Eq. (6), formulated below, is defined as the Jacobian of the SISO controller. In MIMO applications, it is necessary to calculate the exact value of the Jacobian. However, in SISO systems, only the sign of the Jacobian is sufficient for control (Nasseroleslami et al., 2014). The overall weight tuning rule can be calculated from Eq. 7.

(6) $$j=\frac{\partial Z}{\partial \alpha }$$

(7) $$\Delta \,w_{i}=-\eta .\frac{\partial E}{\partial w_{i}}$$

Where *w*_*i*_ is the *i*th neuron weight of the neural controller and η (learning rate) represents the rate of change in weights. Finally, by using the chain derivative rule and combining the relevant equations, Eq. (7) is rewritten as Eq. (10):

(8) $$\frac{\partial E_{e}}{\partial w_{i}}=\frac{\partial E_{e}}{\partial r_{e}}.\frac{\partial r_{e}}{\partial Z}.\frac{\partial Z}{\partial \alpha }.\frac{\partial \alpha }{\partial w_{i}}=-k_{e}.h_{1}.r_{e}.j.\frac{\partial \alpha }{\partial w_{i}}$$

(9) $$\frac{\partial E_{\alpha }}{\partial w_{i}}=\frac{\partial E_{\alpha }}{\partial r_{\alpha }}.\frac{\partial r_{\alpha }}{\partial \alpha }.\frac{\partial \alpha }{\partial w_{i}}=k_{\alpha }.\mathrm{sgn}\,\left(\alpha \right).r_{\alpha }.\frac{\partial \alpha }{\partial w_{i}}$$

(10) $$\Delta w_{i}=\eta \left(k_{e}.h_{1}.r_{e}.j-k_{\alpha }.r_{\alpha }\right).\frac{\partial \alpha }{\partial w_{i}}$$

In these equations, $r_{e}=h_{1}\,e+h_{2}\,\dot{e}+h_{3}\,\int e$and *r*_α_ = *abs*(α). *h*_1_, *h*_2_, *h*_3_, *k*_α_, are assumed as 2, 2, 2, and 0.2, respectively.*k_e_* = 15, 7, 2.5, 1, and 0.1 for levels L1 through L5, respectively. Each muscle is considered as a SISO controller, thus the Jacobian sign would be adequate for control. Each controller-muscle unit minimizes the kinematic error of the node to which it is attached while minimizing its muscle activation. Initial muscle activations were neglected as the muscle forces were adjusted through a feedback strategy.

### Simulations

A total of five simulations (loading cases 1–5), based on the external load distributions and lateral distances of the muscle origins, were considered in this study (Patwardhan et al., 2001; Table 1). Gravitational loads attained their values in 0.2 s. All simulation cases were modeled with and without the muscle/controller. Identical boundary conditions were considered for all simulated cases. The purpose of the controller in this study (i.e., target posture), was to maintain the primary Z-coordinates (minimize lateral deviations between the target and actual positions to remain bounded within 2 mm during the learning process) of the beam, as specified in Table 2. It is noteworthy that the foregoing restrictions on the lateral translations automatically limit any changes in the nodal lateral rotations. The vertical (X direction) displacement of the beam as well as the orientation of the vertebrae were left free to change under the external loads and muscle forces. In addition, in order to investigate the effect of the external load magnitude on the pattern of trunk muscle activations in loading case 2 (Table 1), the muscle forces were recalculated for different external vertical loads (150-750 N) applied at the L1 level.

## Results

### Comparison With the Matched Equilibrium-Based Model

**FIGURE 3:**
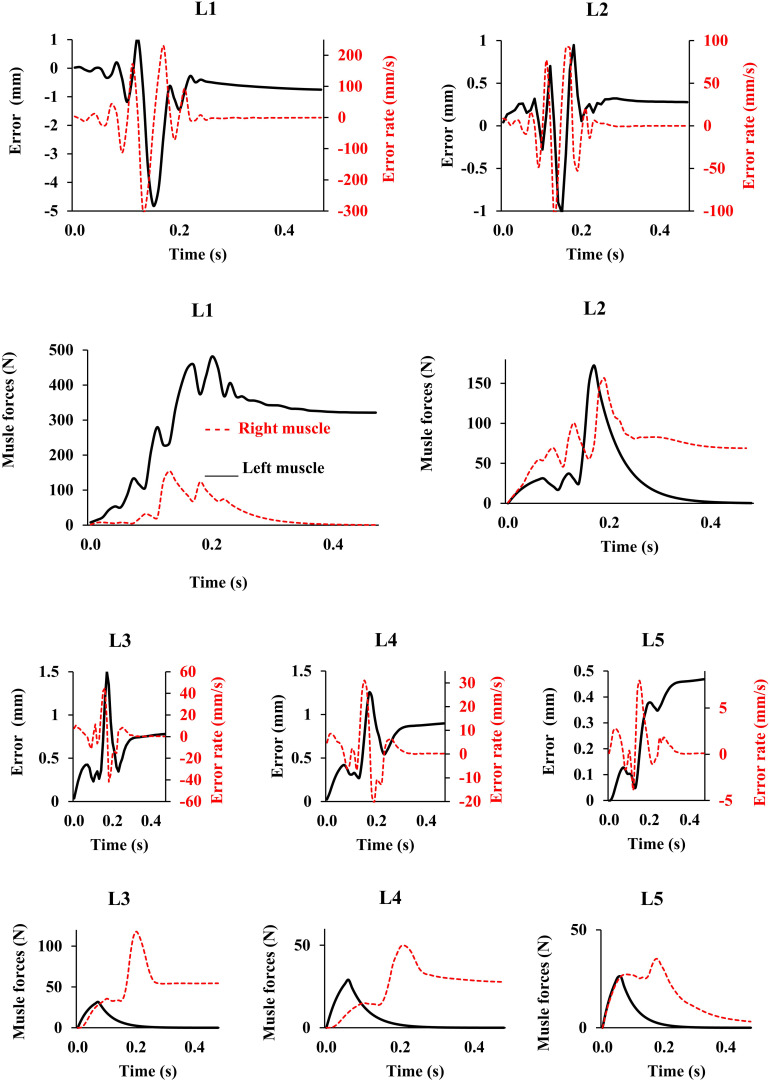
Error, error rate, and muscle forces at different lumbar levels vs. time (up to 0.5 s) for the loading case 1 (with controllers and muscles). Gravitational loads increase from zero to *P*_*i*_ (see Table 1) during 0.2 s. The controller tries to maintain the primary Z-coordinates of beam (Table 2) with a penalty on muscle activation level. For the sake of a clarified visualization, the horizontal axis is cut at 0.5 s while the convergence occurs at ∼20 s.

**FIGURE 4:**
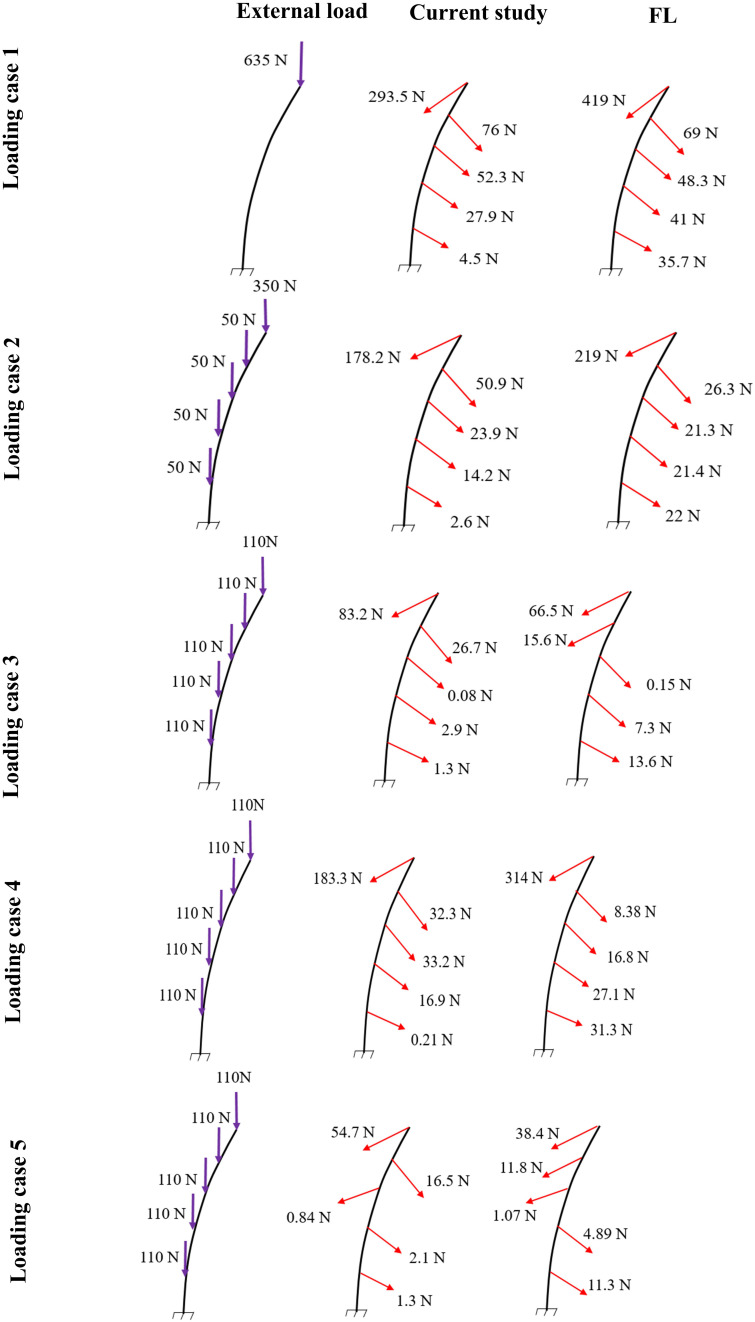
Predicted muscle forces in the current study (middle) as compared to those predicted by a matched equilibrium-based model (Patwardhan et al., 2001) **(right)** for different loading cases **(left)** (Table 1).

In the absence of any controller (i.e., without any muscle activation), the simulated system, expectedly, exhibited large deformations and became unstable. In all five loading cases (Table 1), the controllers in the model successfully learned, over time, to maintain the model close to the target kinematics at equilibrium under the estimated muscle exertions and applied external loads (Figure 3). The actual and target nodal Z-coordinates were different by <0.2 mm during the steady-state condition after 20 s. Initially in the transient period, when the controller was not fully trained, the model deviated slightly (Figure 3) from its target kinematics, and the muscle forces substantially increased. By training the controller, the muscle forces subsequently considerably decreased, and the model reached its steady state. In all five loading conditions, the controllers unilaterally activated only one muscle at each level (i.e., no coactivation). As compared to the matched equilibrium-based model mentioned earlier (Patwardhan et al., 2001), our model predicted similar patterns for muscle forces (i.e., the muscles were activated unilaterally, and their forces decreased going downwards from the upper levels, although generally at lower values (RMSE = ∼41, 16, 12, 44, and 9 N for loading cases 1 through 5, with an overall normalized (to mean) RMSE of 121% for all loading cases) (Figure 4). Consequently, our control-based model predicted smaller compressive loads as compared to its matched equilibrium-based model (Patwardhan et al., 2001; Table 3). However, the equilibrium-based model predicted near zero shear loads in keeping with its own strategy to use the joint reaction forces as an FL (Table 3).

**TABLE 3:** Predicted spinal loads (compression and shear) in the current control-based model as compared to those predicted by a matched equilibrium-based model (Patwardhan et al., 2001) for different loading cases (Table 1).

		**Loading case 2**		**Loading case 3**		**Loading case 4**		**Loading case 5**	
**Load (N)**	**Levels**	**Control**	**Equilibrium**	**Control**	**Equilibrium**	**Control**	**Equilibrium**	**Control**	**Equilibrium**
Shear	L1–L2	−5.1	0.6	−7.6	0.2	−6.4	1.1	−7.4	0.1
	L2–L3	−5.6	−2.0	0.6	−0.9	−5.6	−1.1	0.6	−0.8
	L3–L4	26.2	2.8	16.7	1.6	23.8	3.1	15.3	1.5
	L4–L5	3.6	−0.3	2.5	−0.2	6.5	0.0	2.9	−0.2
	L5–S1	−13.7	0.0	−8.8	0.0	−10.7	0.1	−7.6	0.0
Compression	L1–L2	522	564	191	175	292	423	161	146
	L2–L3	619	638	325	300	433	541	285	267
	L3–L4	690	707	435	410	575	667	396	378
	L4–L5	752	774	547	526	701	802	507	491
	L5–S1	804	837	658	644	811	938	618	606

**Negative shear forces are toward left (Figure 1). Results for Loading case 1 are not reported in the equilibrium-based model; hence no comparison was made.**

### Follower Load (FL) Hypothesis

**FIGURE 5:**
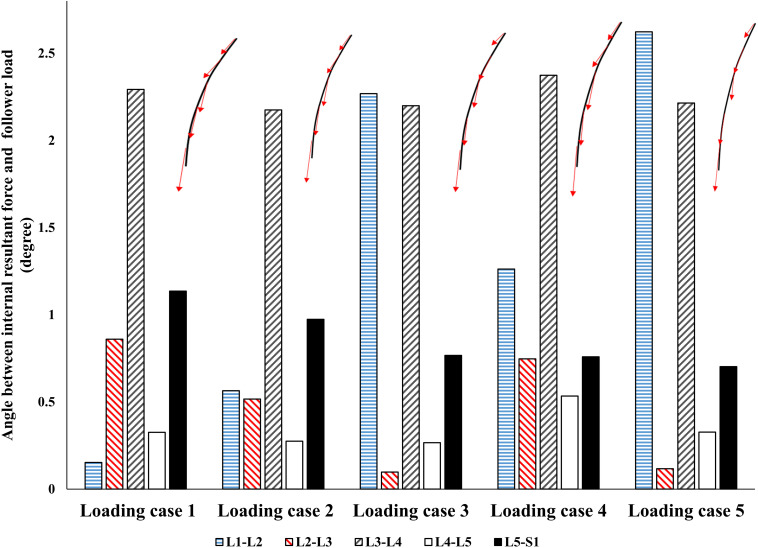
The absolute value of angle between the resultant force on the spine and a hypothetical follower load (FL).

By minimizing the errors between the actual and target nodal Z-coordinates, the control-based model, predicted muscle forces that also generated a near FL on the spine (Figure 5). Regardless of the loading case, the angle between the resultant force on the lumbar spine in our model and an optimal hypothetical FL remained <3°.

### Effect of External Load on Muscle Activations

**FIGURE 6:**
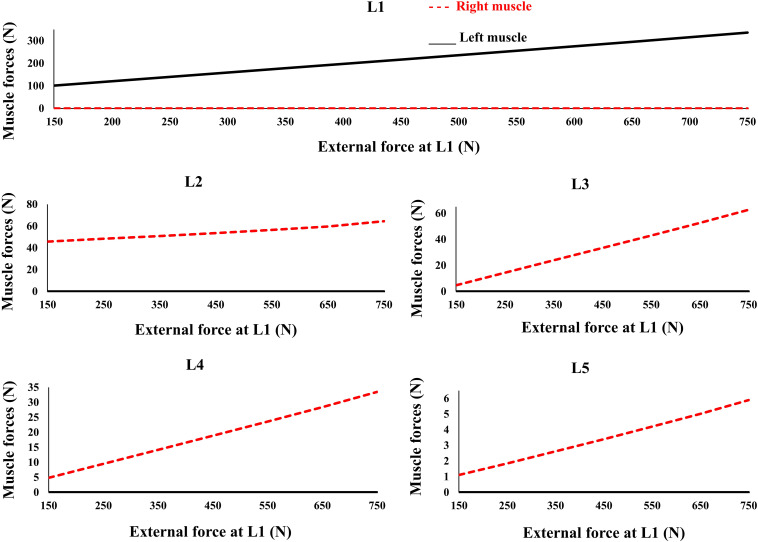
Predicted muscle forces for different external compressive loads acting on the L1 and the distributed loads of 50 N at the L2 to L5 similar to loading Case 2 (Table 1). External force increases from zero to its final value in 0.2 s.

The controllers activated the trunk muscles unilaterally (no bilateral co-activation) regardless of the magnitude of the external loads (Figure 6). Both models produced similar, although not identical activation patterns in the five loading cases. Variation of the muscle forces with the magnitude of external loading within the range simulated at the L1 was found linear.

## Discussion

This study developed a novel geometrically simple control-based model of the lumbar spine and compared its predictions for a slightly bent posture in the frontal plane with those of a geometrically matched equilibrium-based model (Patwardhan et al., 2001). Moreover, the FL concept suggested as an input constraint in the matched equilibrium-based model, as well as, the effect of changes in the externally applied loads on muscle forces, were investigated. The present learning algorithm is classified as a reinforcement-based one, where the controller tends to decrease the defined cost function based on the critic’s signals (Figure 2). The findings indicated that, similar to the equilibrium-based model, the fuzzy neuro-controllers balanced the spine at a given deformed posture using a unilateral muscle force pattern, albeit with generally smaller muscle forces (the sums of muscle forces in our model were smaller by ∼ 159, 40, −11, 132, and −8 N for loading cases 1 through 5, and hence, the L5-S1 compression forces were smaller by ∼ 145, 33, −14, 127, and −12 N for loading cases 1 through 5, respectively). This unilateral muscle activation pattern did not change with the variation of the magnitude of external loads (i.e., the spine was balanced by the controllers without bilateral coactivations). Moreover, for the loading conditions at a slightly laterally bent posture (i.e., quiet standing posture) considered in this study, and consistent with the objective function, the controllers activated the muscles such that the net load on the lumbar spine approached an ideal FL condition. In the future, our control-based approach will be applied to our 3D musculoskeletal model of the spine (Arjmand and Shirazi-Adl, 2006) while simulating various physiological tasks. This model incorporates a realistic geometry of the spine, including ∼80 thoracolumbar muscles, and 6 degrees-of-freedom intervertebral joint with non-linear passive properties. The controllers will aim to determine optimal muscle forces accounting for all the degrees-of-freedom in all anatomical planes. In particular, it would also be interesting to simulate, amongst others, some passive-active injuries and pathological conditions (e.g., altered passive stiffness-muscle coordination/muscle areas).

### Interpretations

Application of the external loads in 0.2 s resulted in an increase in the initial position and velocity beyond those in the target condition (Figure 3). In response, and to maintain equilibrium and stability, the controllers bilaterally and significantly activated the muscles at all levels. Following a transient period with large fluctuations, the controllers succeeded in reducing the errors, such that at the final steady state conditions, the velocity errors completely disappeared, while the position errors diminished to less than 1 mm (Figure 3). At this final static configuration, and in agreement with the matched equilibrium-based model, the controllers activated the muscles unilaterally with no coactivation to balance the spine (Figure 4). The only difference between the two models was observed in loading cases 3 and 5, during which the unilaterally opposite muscles were activated at the L2 level (Figure 4). The control-based model generally balanced the external loads at smaller muscle forces (differences reached ∼159, 40, −11, 132, and −8 N for loading cases 1 through 5). This in in alignment with objective functions minimizing the sum of linear, squared, or cubed muscle forces/stresses, commonly considered in optimization-driven models. While at some levels in the loading cases 3 and 5, our model predicted larger muscular forces, as compared to its matched equilibrium-based model, the sums of muscle forces in these loading conditions, were only moderately larger (11 and 12% increase for loading case 3 and 5, respectively) (Table 3 and Figure 4). This suggests that the cost function used by the CNS to assign forces to muscles may additionally depend on loading conditions and posture. It is to be noted that even smaller total (resultant) spinal loads were estimated in our model when compared to the equilibrium-based model. This highlights the crucial role of our controller (Eq. 5).

Interestingly, without imposing any constraints on the magnitude or direction of muscle or reaction forces in the lumbar spine, a near FL condition was found in various cases (Figure 5). This was in agreement with the matched equilibrium-based model, which constrained activation in muscles to generate an FL on the spine at all levels. It appears, therefore, that the controllers (i.e., the CNS) learned to balance and stabilize the spine by generating conditions approaching that under an FL. This is also in agreement with findings from another detailed musculoskeletal equilibrium-based model of the spine, in which the muscle forces were predicted to create compressive FLs on the spine during a quiet standing posture (Han et al., 2011). The outcome in internal loading is also consistent with the minimization of changes in horizontal translations. Moreover, unlike the equilibrium-based model which predicted no shear loads on the spine, small spinal shear loads were predicted in our model (Table 3). The structure and nature of the constitutive components of the objective function in our model (Eq. 5) allow for diverse simulation possibilities to explore the competing goals of the system toward emulating the sophisticated physiological system and its intricate strategies. The addition of more state variables can be another intriguing motivation for future investigation.

The recruitment of trunk muscles has been shown to be strongly direction dependent (Nussbaum et al., 1995; Hadizadeh et al., 2014; Sedaghat-Nejad et al., 2015; Eskandari et al., 2016). In quasi-static conditions the emergent synergies responsible for a direction of external load will be linearly scaled. The invariance in set of activated muscles under varying magnitude of external load (Figure 6) is in line with the theories of using muscle synergy in multiple muscle systems across the cost functions (Moghadam et al., 2013; Eskandari et al., 2016). Future studies must test this in more physiological models with realistic posture/loading and non-linear properties. Future studies can also benefit by incorporating more physiologically based detailed architectural/geometrical muscle models and structure/function data obtained from neuroimaging studies.

### Limitations

**FIGURE 7:**
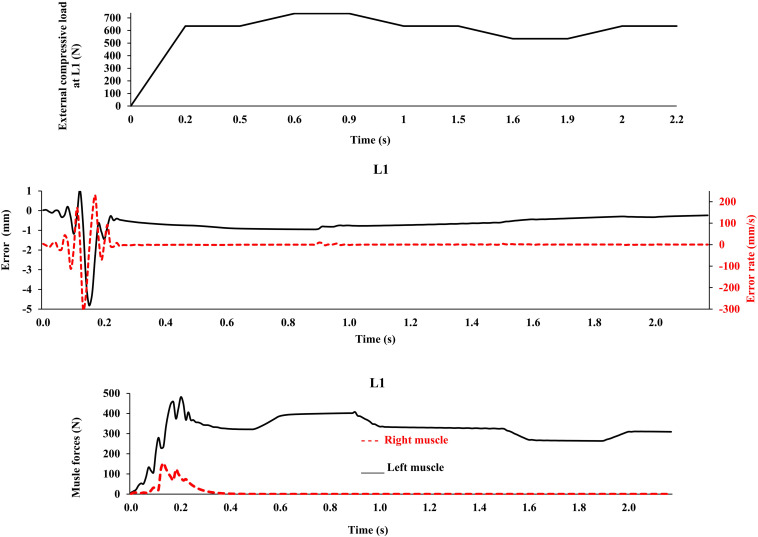
External compressive force, error, error rate and muscle forces at L1 vs. time (up to 2.2 s) for the loading case 1 under application of −100 N and 100 N vertical force at L1 during [0.5 s, 1 s] and [1.5 s, 2 s] time intervals, respectively, in order to model perturbations (with controllers and muscles). The controller tries to maintain the primary Z-coordinates of beam (Table 2) with a penalty on muscle activation level.

This model was idealized in terms of the geometry of the active-passive tissues, material properties, and loading conditions in the frontal plane, as we primarily aimed to (1) implement a novel bio-inspired control strategy that mimics the adaptive mechanism of the CNS and (2) compare its predictions with an existing matched equilibrium-based model. As the current model was idealized based on simplifying assumptions in terms of the geometry of the spine, loading, boundary conditions, and musculature, caution should be exercised when extrapolating results to clinical applications. The maximal force in all muscles was considered to be 800 N, in order to accommodate large fluctuations in muscle forces during the transient period (Figure 3). In the final steady-state, however, much smaller muscular forces were estimated (Figure 4). Non-zero muscle pre-activation values (initial values) could subdue the fluctuations observed in the transient state. While the stability was not formally examined in our model, different perturbations (e.g., the addition of a moment at the L1 and the reduction of Young’s modulus of the beams; Nasseroleslami et al., 2014) did not cause instability, as the controllers prevented large deformations and maintained the final steady-state position. For example, Figure 7 depicts the model response under a perturbation, where the addition and removal of a 100 N load to impose external compression of 635 N at L1 for a duration of 0.5 s, caused the muscle force to appropriately rise and fall, respectively, to maintain the required objective posture (*Z* coordinates). The error terms, which approached nil at the end of the 20 s simulation, are not shown in Figure 7 for clarity. The closed loop response could include multiple loops with varying gains and time delays (Zeinali-Davarani et al., 2008). We have neither considered the spindle nor the reflexive responses in the feedback loop, and we have not used an internal model to assist with the initial exploration of activation selection (Dariush et al., 1998; Shadmehr and Mussa-Ivaldi, 2012), all warranting future investigation. The objective function should be designed considering stability in the Lyapunov sense, while setting the performance criterion to maintain the system within the safe normal physiological limits of the passive and active spinal structures. This provides an envelope with margins of safety to avoid pain, discomfort, muscle fatigue, instability and ultimately failure/injury.

## Conclusion

This work presents a new method to estimate muscle forces using a control-based FE model of the lumbar spine. The model incorporates a control strategy that mimics the adaptive mechanism of the CNS to adjust muscle forces. Steady state muscle forces have similar patterns to a geometrically matched equilibrium-based model and spine reaction forces resemble a FL on the spine. Additionally, controllers linearly scale muscle forces in a specific loading condition with varying magnitude of external load. The phenomenon of FL is the predicted behavior of this adaptive neuro-fuzzy control system and not the explicit objective of the mathematical theory or conjecture. That creates a fertile paradigm to consider clinical ideas (i.e., spinal injuries and/or fusion) to be investigated in future studies with a more detailed architecture for muscles under more general loading conditions during daily activities at work, leisure and sport.

## Data Availability Statement

All datasets generated for this study are included in the article/supplementary material.

## Author Contributions

All authors listed above have made substantial contributions to the conception and design of the study, analysis and interpretation of data, preparing the manuscript, and have read the final approval of the version to be submitted and listed on the title page have read the manuscript and also attest to the validity and legitimacy of the data and its interpretation, and agreed to its submission.

## Conflict of Interest

The authors declare that the research was conducted in the absence of any commercial or financial relationships that could be construed as a potential conflict of interest.
